# Determining classes of food items for health requirements and nutrition guidelines using Gaussian mixture models

**DOI:** 10.3389/fnut.2023.1186221

**Published:** 2023-10-13

**Authors:** Yusentha Balakrishna, Samuel Manda, Henry Mwambi, Averalda van Graan

**Affiliations:** ^1^Biostatistics Research Unit, South African Medical Research Council, Durban, South Africa; ^2^School of Mathematics, Statistics and Computer Science, University of KwaZulu-Natal, Pietermaritzburg, South Africa; ^3^Department of Statistics, University of Pretoria, Pretoria, South Africa; ^4^Biostatistics Research Unit, SAFOODS Division, South African Medical Research Council, Cape Town, South Africa; ^5^Division of Human Nutrition, Department of Global Health, Stellenbosch University, Cape Town, South Africa

**Keywords:** food composition database, nutrient table, mixture model, clustering, classification, nutritional content

## Abstract

**Introduction:**

The identification of classes of nutritionally similar food items is important for creating food exchange lists to meet health requirements and for informing nutrition guidelines and campaigns. Cluster analysis methods can assign food items into classes based on the similarity in their nutrient contents. Finite mixture models use probabilistic classification with the advantage of taking into account the uncertainty of class thresholds.

**Methods:**

This paper uses univariate Gaussian mixture models to determine the probabilistic classification of food items in the South African Food Composition Database (SAFCDB) based on nutrient content.

**Results:**

Classifying food items by animal protein, fatty acid, available carbohydrate, total fibre, sodium, iron, vitamin A, thiamin and riboflavin contents produced data-driven classes with differing means and estimates of variability and could be clearly ranked on a low to high nutrient contents scale. Classifying food items by their sodium content resulted in five classes with the class means ranging from 1.57 to 706.27 mg per 100 g. Four classes were identified based on available carbohydrate content with the highest carbohydrate class having a mean content of 59.15 g per 100 g. Food items clustered into two classes when examining their fatty acid content. Foods with a high iron content had a mean of 1.46 mg per 100 g and was one of three classes identified for iron. Classes containing nutrient-rich food items that exhibited extreme nutrient values were also identified for several vitamins and minerals.

**Discussion:**

The overlap between classes was evident and supports the use of probabilistic classification methods. Food items in each of the identified classes were comparable to allowed food lists developed for therapeutic diets. This data-driven ranking of nutritionally similar classes could be considered for diet planning for medical conditions and individuals with dietary restrictions.

## 1. Introduction

The study of single nutrients in food items has played an important role in our understanding of the basic causes and treatment strategies of nutrition-related diseases (1). Establishing the relationships between specific nutrients and food items and determining the association between specific nutrient intakes and diseases, may help with the interpretation of dietary patterns found in a population and the explanation of the association between dietary patterns and disease (2). In addition, a reasonable first step toward the development of food-based dietary guidelines (FBDGs) is identifying the food sources of the nutrient of interest. This information can be ascertained from food composition databases (FCDBs) and understanding food items and their nutrients promotes a basic knowledge of nutrition amongst the population (3). The analysis of dietary patterns is dependent on the categorization of food items but the rules determining this categorization, which are based on conceptual and compositional similarity, are not always well-defined (2).

The need to group foods by nutritional content was recognized by Khan (4) who proposed categorizing foods as having a either a low, medium or high specific nutrient content to assist dietitians with food recommendations. However, the proposed category thresholds were suggestive and a more rigorous method of determining the thresholds was needed. More recently, a more suitable, data-driven categorization was proposed, using *k*-means clustering to group foods by nutrient content (5). Other methods that have been used to classify food items are hierarchical clustering, principal component analysis (PCA), factor analysis and fuzzy clustering (6). Thus, employing statistical clustering methods to food composition data can produce objectively determined classes. A previous study (7) applied PCA to food composition data to identify nutritionally similar groups. However, evaluating similar food items through PCA does not account for the uncertainty in assigning food items to classes. In addition, while food items were able to be grouped by overall nutritional similarity, food items were unable to be ranked by the level of a specific nutrient content. The ability to rank food items by the level of nutrients is essential for creating food lists for therapeutic diets. Some common therapeutic diets that involve nutrient modification are renal diets for the management of chronic kidney disease (8) and low carbohydrate diets for the management of diabetes (9).

A recent review has shown that mostly centroid-based and hierarchical clustering techniques have been applied to food composition data (6) but mixture models have yet to be investigated in this context. The application of mixture models to identify dietary patterns in food consumption studies has shown advantages over nonparametric approaches (10, 11). Nonparametric approaches result in classes wherein each food item belongs exclusively to one class, thus assuming that the classification uncertainty is zero. However, if arbitrary thresholds existed to separate low and high nutrient content foods, there is a weak separation between food items containing nutrient levels that are near the threshold. Mixture models accommodate for this uncertainty by measuring the probability of class membership, which takes values between zero and one (10). With probabilistic clustering, the focus is not on whether a food is in a class, but rather to what extent it is associated with that class (12). The consideration of the uncertainty in determining nutritional classes allows for greater precision and reduced allocation bias (13).

Probabilistic clustering or distribution-based clustering assumes that the nutrient values are generated by a mixture of probability distributions and that each distribution forms a class. Each food item is assigned a probability of class membership (these being the posterior probabilities), thus supporting multiple class membership and also the assignment of outliers to classes. The most popular algorithm of this approach is the Gaussian mixture model (GMM). For a dataset of *n* food items that one wants to classify into *k* compositionally similar groups, the GMM assumes that the overall nutrient content distribution consists of a mixture of *k* Gaussian (normal) distributions. In this study, we apply univariate GMMs to food composition data to determine classes that contain similar levels of specific nutrients and to allow for the estimation of the class membership probabilities for each food item.

## 2. Materials and methods

### 2.1. Data

The 2017 SAFCDB (11) contains nutritional information for 1,667 food items and 169 food components (hereon termed ‘nutrients’). The compilation of food composition data for the SAFCDB comprises various number of data sources ranging from national projects involving direct methods and indirect methods, to the sourcing of scientific literature, certificate of analyses and product nutritional information from various data generators.

Of the 169 food components, we selected the most common nutrients with the least amount of missing values for inclusion. We also considered nutrients that were non-collinear. For example, since total carbohydrate is the sum of available carbohydrate and dietary fibre, available carbohydrate and dietary fibre were included instead of total carbohydrate. Using these criteria, we selected 28 nutrients (nine macronutrients, nine minerals and ten vitamins) for analysis and included food items (*n* = 971) which had non-missing nutrient information for all 28 nutrients.

For each of the 28 nutrients, each of the 971 food items had either a known nutrient value, a zero nutrient value or a trace value. Food items with a zero nutrient value for a particular nutrient are excluded from the univariate GMM analysis since we are interested in classifying only food items known to have the nutrient of interest. Trace values were imputed with half the limit of detection for each nutrient (14). Thus, only food items containing either a known nutrient value or trace value are included in the analysis. Extreme nutrient values were retained in the dataset. Raw food items, cooked food items and combined dishes (where nutrient composition has been calculated using standard recipes) from various food groups were included in the analysis (Table 1). All nutrient values were expressed per 100 g edible part.

**Table 1:** Number of food items analyzed by food group.

Food group	*n* (%)
Cereals and cereal products	195 (20.08)
Vegetables	245 (25.23)
Fruit	132 (13.59)
Legumes and legume products	26 (2.68)
Nuts and seeds	20 (2.06)
Milk and milk products	41 (4.22)
Eggs	27 (2.78)
Meat and meat products	120 (12.36)
Fish and seafood	36 (3.71)
Fats and oils	26 (2.68)
Sugar, syrups and sweets	17 (1.75)
Soups, sauces, seasonings and flavorings	30 (3.09)
Beverages	27 (2.78)
Infant and paediatric feeds and foods	10 (1.03)
Therapeutic/special/diet products	7 (0.72)
Miscellaneous	12 (1.24)
Total	971 (100)

### 2.2. Methods

#### 2.2.1. Univariate Gaussian mixture model

In the case of food composition data, the univariate Gaussian mixture model assumes that the nutrient content values arise from a mixture of two or more Gaussian distributions. Each Gaussian distribution represents a class of food items. Since Gaussian distributions can be described by the mean and variance, the means and variances for each class of food items can be estimated.

The means and variances for each class of food items can be estimated via an iterative process called the Expectation–Maximization (EM) algorithm (15). Since we do not know the means and variances for each class of food items beforehand, we begin with an initial guess for each and iterate between an expectation step (E-step) and a maximization step (M-step). In the E-step, we calculate the probability that a food item belongs to a specific class. In the M-step, we update the mean and variances for each class, based on the probabilities calculated in the expectation step. The steps are repeated until there are no significant changes in either the means and variances or the log-likelihood (how well the model fits the data). The mathematical definitions of the univariate GMM follow.

Suppose that $x_{ij}$ is the amount of nutrient j for food item i ( i=1,2,…,971;j=1,2,…,28) . We assume that the nutrient value $x_{ij}$ arises from a mixture composed of k unobserved classes. Formally, $x_{ij}$ is a sum of class-specific nutrient distributions as


$$p\left(x_{ij}\right)=\overset{K}{\underset{k=1}{\sum }}p\left(x_{ij}|z_{ij}=k\right)p\left(z_{ij}=k\right)$$

$$=\overset{K}{\underset{k=1}{\sum }}\pi_{k}p_{k}\left(x_{ij}\right)$$

where K is the number of classes, $z_{ij}=1,2,\ldots ,k,\ldots ,K$ indicates the class for $x_{ij}$ , $p_{k}\left(x_{ij};\theta_{k}\right)$ is the probability distribution for class k with parameter vector $\theta_{k}$ and $\pi_{k}$ is the proportion of food items that belong to class k such that


$$0\leq \pi_{k}\leq 1$$

and


$$\overset{K}{\underset{k=1}{\sum }}\pi_{k}=1$$

Assuming $p_{k}\left(x_{ij}\right)=N\left(\mu_{k},\sigma_{k}^{2}\right)$ , then $p_{k}\left(x_{ij}\right)$ follows a Gaussian distribution and $p\left(x_{ij}\right)$ becomes a Gaussian mixture distribution. Thus, for the univariate GMM


$$z_{ij}\sim \mathrm{Cat}\left(\pi \right)$$

$$x_{ij}|z_{ij}=k\sim N\left(\mu_{k},\sigma_{k}^{2}\right)$$

where π is the vector of proportions, $\mu_{k}$ is the mean nutrient content for class k and $\sigma_{k}$ is the associated standard deviation for class k .

The EM algorithm can be utilized when we need to conduct a maximum likelihood estimation of parameters in the presence of missing data or latent variables. The E- and M-steps for the univariate GMM are outlined below.

#### 2.2.2. The E-step

Calculate the responsibilities $\gamma_{iz}$ (posterior probabilities) for the *i*th food item and *z*th class:


$$\gamma_{iz}=\frac{\pi_{z}\left(\frac{1}{\sigma_{z}\sqrt{2\pi }}\exp\left(-\frac{1}{2\sigma_{z}^{2}}{\left(x_{i}-\mu_{z}\right)}^{2}\right)\right)}{\sum_{k=1}^{K}\pi_{k}\left(\frac{1}{\sigma_{k}\sqrt{2\pi }}\exp\left(-\frac{1}{2\sigma_{k}^{2}}{\left(x_{i}-\mu_{k}\right)}^{2}\right)\right)}$$

#### 2.2.3. The M-step

Calculate the new parameters $\mu_{z}^{*}$ , $\sigma_{z}^{*}$ , and $\pi_{z}^{*}$ via maximization using


$$\mu_{z}^{*}=\frac{\sum_{i=1}^{N}\gamma_{iz}x_{i}}{\sum_{i=1}^{N}\gamma_{iz}}$$

$$\sigma_{z}^{*}=\sqrt{\frac{1}{\sum_{i=1}^{N}\gamma_{iz}}\overset{N}{\underset{i=1}{\sum }}\gamma_{iz}{\left(x_{i}-\mu_{z}^{*}\right)}^{2}}$$

$$\pi_{z}^{*}=\frac{\sum_{i=1}^{N}\gamma_{iz}}{N}$$

The EM algorithm begins with initialization and is iterated until convergence of the parameters or log-likelihood is reached (16).

#### 2.2.4. Statistical analysis

After examining the distributions for each nutrient, we aimed to fit a univariate GMM for each natural log-transformed nutrient. ‘Moisture’ was kept on the original scale. We used the ‘mclust’ (17) and ‘mixtools’ (18) R packages to fit the models. The steps followed are outlined below. For each of the 28 nutrients:

1. We determined the optimal number of classes to fit using quantiles to initialize the EM algorithm. Ten GMMs were fitted in succession for *k* (the number of classes) ranging from 1 to 10 and the Bayesian Information Criterion (BIC) (19) was computed for each model. The *k* that minimized the BIC was selected as the optimal number of classes.
2. We used the EM algorithm with random initialisation to fit the GMM with the optimal *k*. To avoid local optima, the model was fitted 10 times and the model with the highest log-likelihood was selected. Convergence was declared when the change in the observed log-likelihood increased by less than $10^{-8}$ .
3. The parameter estimates for the mean ( μ ), standard deviation ( σ ) and proportion ( π ) from the selected model were recorded and the GMM density function was plotted.
4. Food items were assigned to classes based on their highest estimated probability of class membership. The class validity of the GMM solutions was assessed using the Davies-Bouldin (DB) (20) index and silhouette coefficient (21).

The DB index measures the average separation between each class and its next nearest class. The index is bounded between zero and infinity with values closer to zero indicating a better partitioning. The silhouette coefficient measures how similar an observation is to observations in its own class (compactness) compared to observations in other classes (separation). The silhouette coefficient is bounded between −1 and 1, where negative values indicate incorrect classifications, values close to 1 indicate highly dense classifications and scores around zero indicate overlapping classifications (observations lying between two classes). Scores greater than 0.5 are generally desirable for good classifications (22).

## 3. Results

### 3.1. Model selection

The BIC was compared for the 1- to 10-class GMMs. The most frequent model selected was the two-class model (n = 14/28) followed by the four-class model (*n* = 6/28). The highest number of classes was found when food items were grouped based on sodium content and niacin content with five and seven classes, respectively. Plant protein, calcium and vitamin B_6_ were best described by a single class, that is, the univariate normal model.

### 3.2. Identified classes

**Figure 1:**
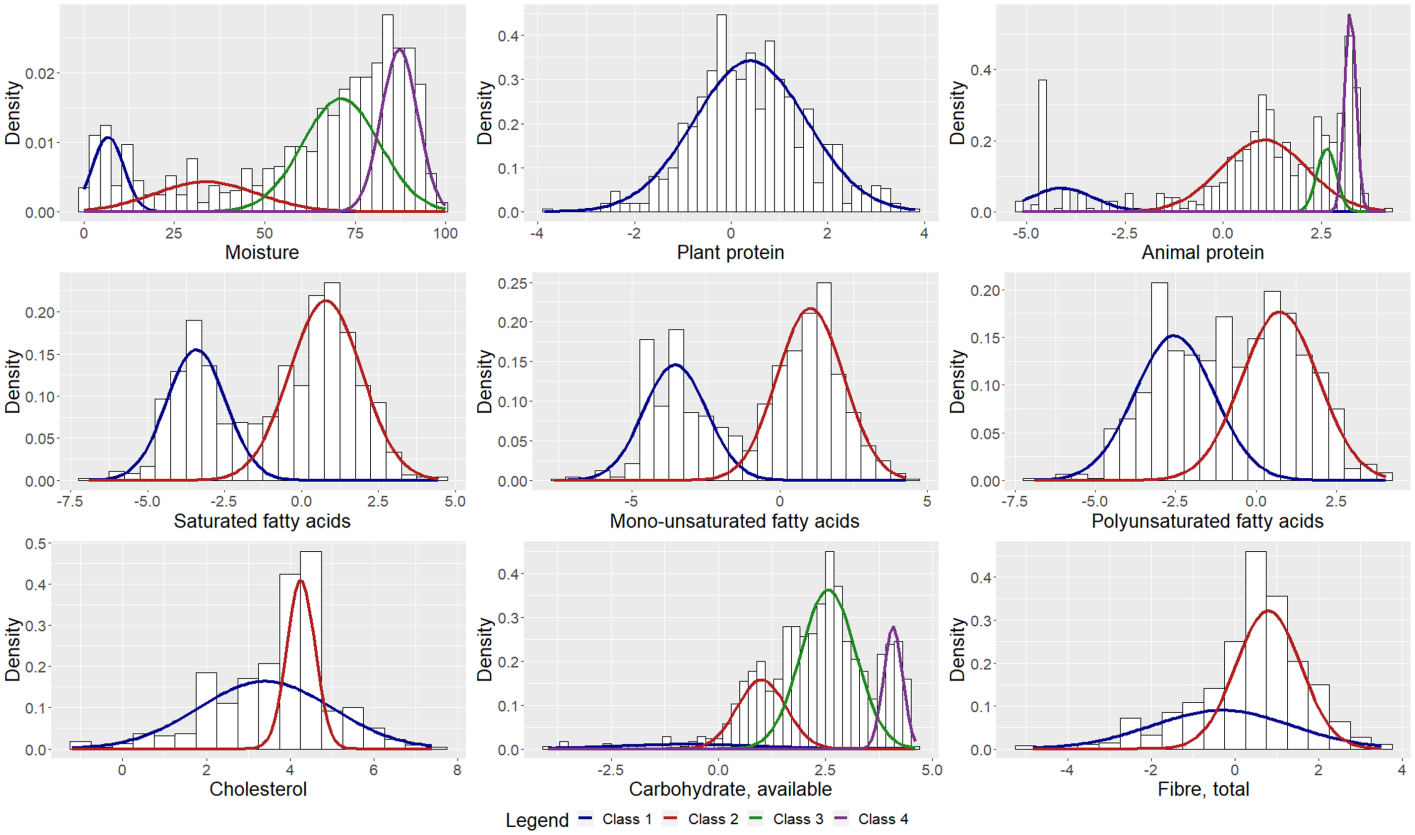
Univariate Gaussian mixture model for macronutrients.

**Figure 2:**
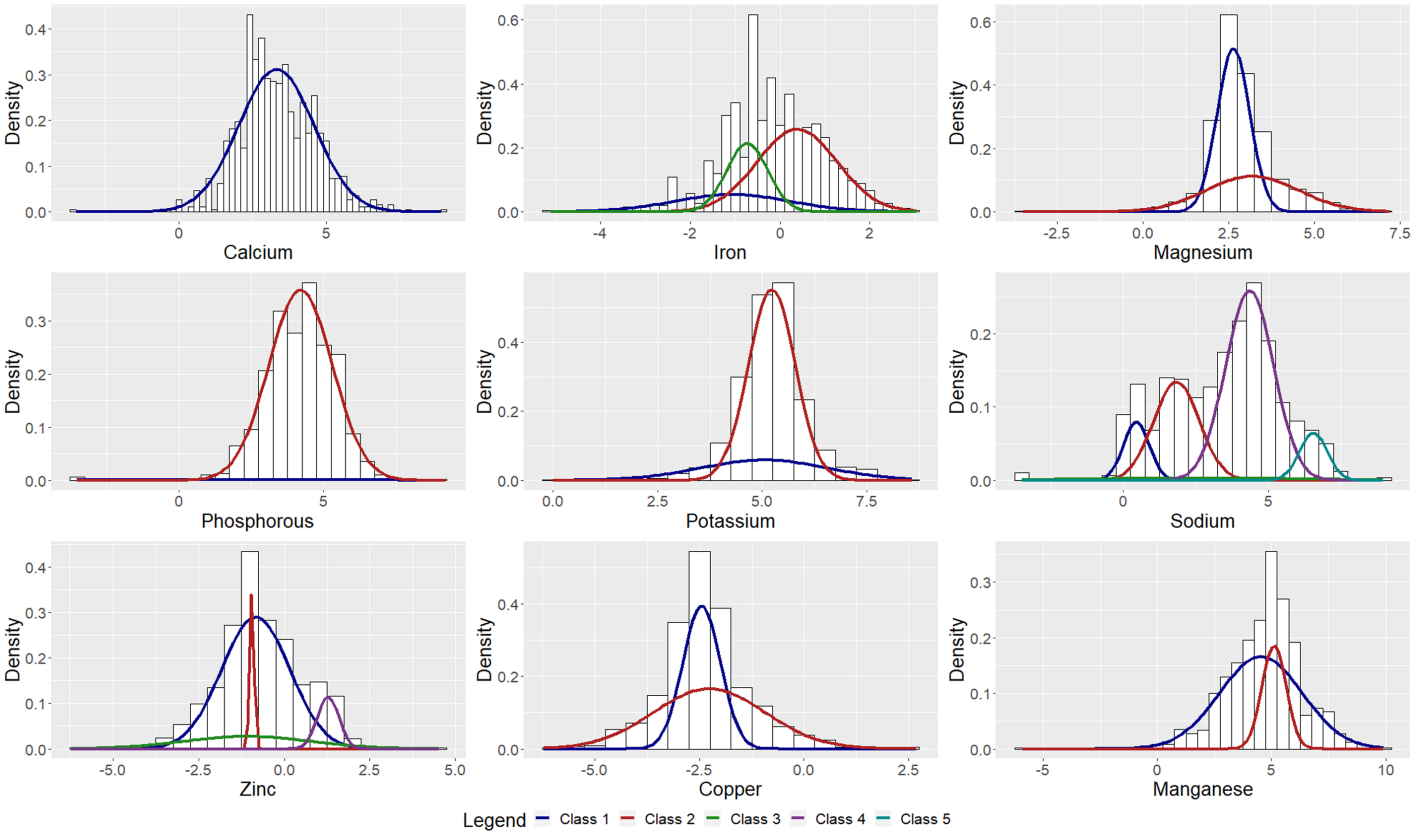
Univariate Gaussian mixture model for minerals.

**Figure 3:**
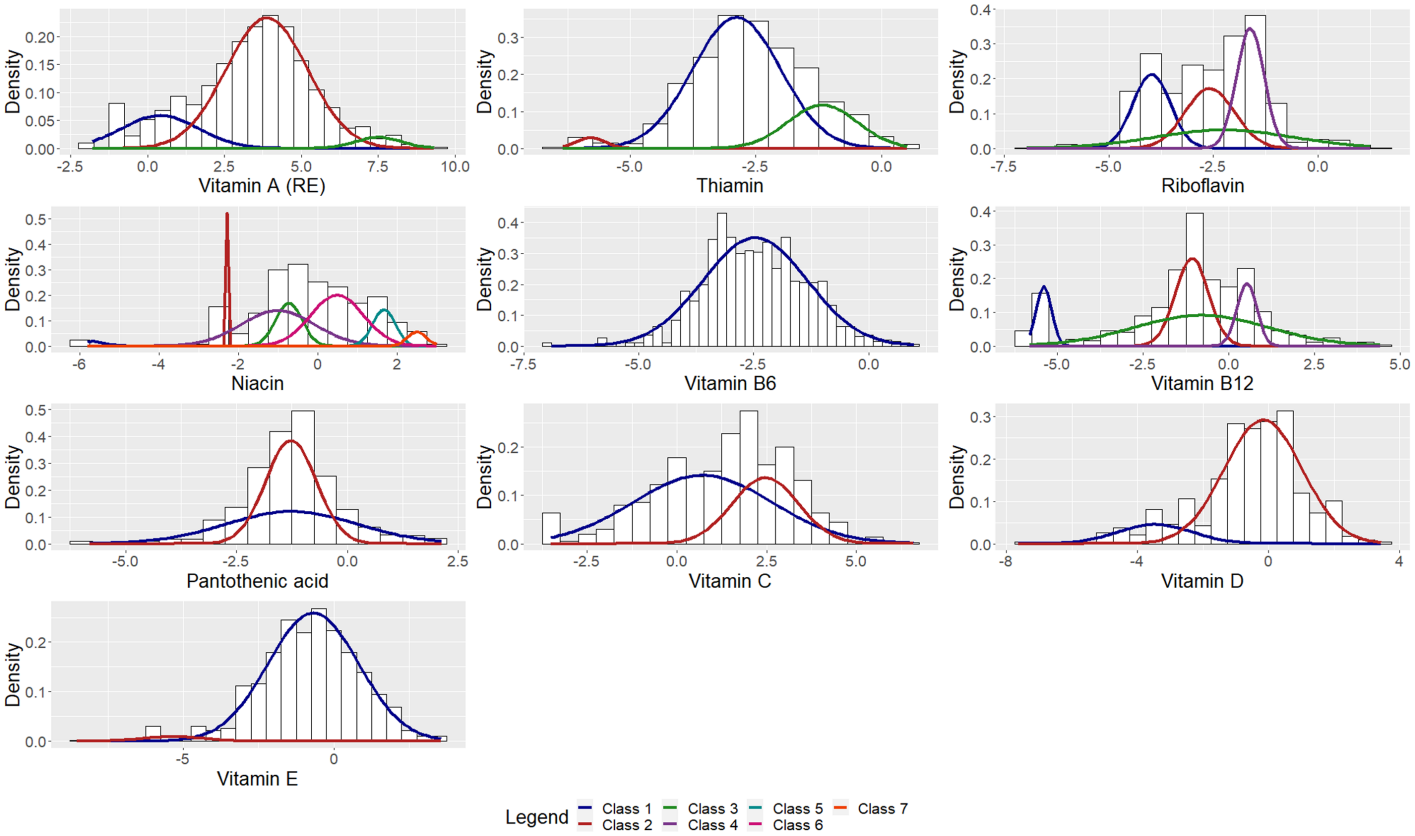
Univariate Gaussian mixture model for vitamins.

The parameter estimates corresponding to the classes are presented in Table 2. Figures 1–3 depict each nutrient-based classification, which can be described as a mixture of Gaussian distributions. Hence, each Gaussian distribution on the plots represents a class.

**Table 2:** Parameter estimates for the univariate Gaussian mixture model^§^.

Nutrient	*N*	Class 1			Class 2			Class 3			Class 4			Class 5			Class 6			Class 7		
		%	Mean	SD	%	Mean	SD	%	Mean	SD	%	Mean	SD	%	Mean	SD	%	Mean	SD	%	Mean	SD
Moisture (g)	964	12	6.68	4.33	15	33.8	14.35	43	71.1	10.65	29	87.2	5.03									
Plant protein (g)	749	100	1.49	1.17																		
Animal protein (g)	487	13	0.02	0.81	55	2.86	1.1	11	13.87	0.24	20	25.53	0.14									
Saturated fatty acids (g)	956	37	0.03	0.96	63	2.2	1.17															
Mono-unsaturated fatty acids (g)	955	39	0.03	1.06	61	2.83	1.12															
Polyunsaturated fatty acids (g)	957	47	0.08	1.23	53	2.1	1.2															
Cholesterol (mg)	434	65	30.3	1.59	35	70.81	0.34															
Carbohydrate, available (g)	879	4	0.48	1.64	22	2.75	0.56	58	13.07	0.64	15	59.15	0.22									
Fibre, total (g)	752	38	0.73	1.67	62	2.23	0.77															
Calcium (mg)	961	100	27.39	1.28																		
Iron (mg)	965	18	0.34	1.31	58	1.46	0.89	25	0.48	0.46												
Magnesium (mg)	960	62	14.01	0.48	38	24.29	1.35															
Phosphorous (mg)	959	2	6.11	3.55	98	66.69	1.1															
Potassium (mg)	963	23	156.02	1.53	77	186.79	0.56															
Sodium (mg)	959	9	1.57	0.45	27	6.23	0.79	3	13.07	3.75	54	78.26	0.83	8	706.27	0.48						
Zinc (mg)	962	72	0.44	0.99	6	0.39	0.06	13	0.34	1.87	9	3.6	0.32									
Copper (mg)	958	44	0.09	0.45	56	0.1	1.34															
Manganese (μg)	957	75	93.69	1.81	25	165.67	0.53															
Vitamin A (RE) (μg)	817	18	1.51	1.25	78	48.42	1.33	4	1844.57	0.76												
Thiamin (mg)	954	77	0.06	0.87	2	0.003	0.29	20	0.31	0.7												
Riboflavin (mg)	960	24	0.02	0.45	26	0.08	0.59	22	0.11	1.68	28	0.2	0.33									
Niacin (mg)	954	1	0.003	0.33	5	0.1	0.02	14	0.48	0.33	33	0.36	0.94	10	5.26	0.28	33	1.62	0.67	3	12.06	0.24
Vitamin B_6_ (mg)	952	100	0.08	1.14																		
Vitamin B_12_ (μg)	487	10	0.005	0.22	32	0.34	0.48	45	0.45	1.97	13	1.7	0.29									
Pantothenic acid (mg)	954	45	0.29	1.49	55	0.28	0.57															
Vitamin C (mg)	721	68	2.03	1.93	32	11.7	0.93															
Vitamin D (μg)	471	13	0.03	1.14	87	0.85	1.19															
Vitamin E (mg)	924	98	0.51	1.5	2	0.005	1.05															

§ Mean estimates are presented on its original scale per 100 g. Standard deviation (SD) estimates are presented on the natural-log scale. The percentage (%) of food items belonging to the class is also reported.

Five classes of food items were identified when classifying food items by sodium content and the mean sodium content of the classes ranged from 1.57 mg to 706.27 mg per 100 g (Table 2). Food items identified as having the highest sodium content were bread, potato crisps, breakfast cereals, canned vegetables, dehydrated potato mash, milk powders, processed meat, canned/cured/smoked fish, butter, margarine, mayonnaise and packaged soup mix (Table 3). Grouping foods by their available carbohydrate content resulted in four identified classes (Table 2). Class 4 contained foods with the highest mean available carbohydrate content of 59.15 g per 100 g and consisted of baked goods, starchy vegetables, and sugar and sweets (Table 4). Food items grouped by their fatty acid content were found to consist of two classes for each of the fatty acids, suggesting that food items could naturally be grouped into having either a low or a high fatty acid content. Food items associated with having a high fatty acid content were baked goods, fried foods, nuts and seeds, dairy products, eggs, meat products, caviar, high-fat fish and fats and oils (Table 5). Three classes of food items were identified when the grouping was based on iron content. Class 2 had the highest mean iron level of 1.46 mg per 100 g and contained mainly wheat products, dehydrated raw vegetables, green vegetables, beetroot, mushroom, dried fruit, legumes, nuts and seeds, milk powder with added iron, eggs, meat (excluding white meat chicken and veal) and certain seafood (Table 6).

**Table 3:** Food items within the identified sodium classes.

Class	Class 1	Class 2	Class 3		Class 4	Class 5
Class description	Low content	Moderately-low content	Extremely low content	Extremely high content	Moderately-high content	High content
Food group						
Cereals and cereal products	Cooked maize meal porridges, cooked white rice, cooked oats, wheat flour, cooked pasta, uncooked semolina, roti	Cooked wheat, cooked egg noodles, cooked, brown rice, brown rice flour, cooked barley, raw maize meal, wheat germ, wheat flour, cooked wholewheat pasta			Baked goods, pasta dishes	Bread, potato crisps, breakfast cereals, self-raising wheat flour
Vegetables	Squash, potato, melon, boiled pumpkin	Bamboo shoots, green beans, tomato, baby marrow squash, brinjal, leaves, peas, mushroom, Brussels sprouts, onion, white-fleshed sweet potato, cauliflower, cabbage	Asparagus soup and boiled mangetout		Beetroot, vegetables cooked with margarine, dehydrated raw vegetables, carrots, leaves, baby sweetcorn, celery, canned vegetables	Canned baby sweetcorn, canned asparagus, canned sauerkraut, dehydrated potato mash, spinach, dehydrated cauliflower, canned olives
Fruit	Apple, banana, gooseberry, grapes, grapefruit juice, guava, lemon juice, mango, naartjie juice, orange juice, orange, pineapple, sour plum, prickly pear, raspberry, rhubarb, youngberry, date, granadilla, kiwifruit, lime, marula, medlar, mineola, nectarine, dried peach, dried prune	Canned fruit, stewed fruit, dried fruit, prunes, dates, pawpaw, figs, cherries, plums, peaches, rhubarb stems, strawberry, watermelon, kumquat, avocado, grapefruit, lemon, litchi, pear			Melon, raisins, fruit mincemeat, dried apple, candied orange/lemon peel, glazed cherry	
Legumes and legume products	Dried beans, cooked split peas, cooked lentils	Cooked rice and lentils dish, raw lentils, raw split peas, tofu, cooked beans, cooked chickpeas			Bean dishes, raw chickpeas, lentil dishes	
Nuts and seeds	Almonds (unsalted, blanched), pistachios, chestnuts, coconut, pine nuts, walnut	Unsalted peanuts, macadamia nuts, sunflower seeds, Brazil nuts, cashew nuts, unblanched almonds			Sesame seeds, desiccated coconut, salted peanuts	
Milk and milk products					Milk, yoghurt, custard, cottage cheese	Cheese, milk powders (low-fat, skim, added vitamins)
Eggs					Eggs	Dried egg
Meat and meat products					Meat	Processed meat
Fish and seafood				Fish biltong, anchovy	Fish, oyster, tuna, crab, mussels	Shrimp/prawn, rollmop/pickled herring, caviar, smoked fish, canned sardine
Fats and oils	French salad dressing, butter ghee, olive oil	Pressurized cream			Salad dressing, cream	Butter, margarine, mayonnaise
Sugar, syrups and sweets	Sugar	Honey, dark chocolate, jam/marmalade, jelly (with fruit)			Chocolate, icing, molasses	
Soups, sauces, seasonings and flavorings		Curry sauce, soup mix (with beef and vegetables)			Sauces and soups	Soup (packet mix)
Beverages		Fruit juices, fruit nectars			Milk beverages	
Infant and paediatric feeds and foods					Infant feeds	
Therapeutic/special/diet products					Therapeutic powders	
Miscellaneous	Tea, spirits	Vinegar, wine, liqueur, sherry, tea		Baking powder	Liqueur with cream	

**Table 4:** Food items within the identified available carbohydrate classes.

Class	Class 1	Class 2	Class 3	Class 4
Class description	Extremely low content	Low content	Moderate content	High content
Food group				
Cereals and cereal products			Milk tarts, white rice, pancakes, puddings, pasta dishes, soft and stiff maize meal porridge, scones	Raw maize meal, rice flour, wheat flour, potato flour, oats, cookies, cakes, bread, breakfast cereals
Vegetables	Rhubarb	Cucumber, leaves, marrow squash, spinach, broccoli, cabbage, cauliflower, Brussels sprouts, mushroom, brinjal, tomato, avocado, rhubarb stems, olives	Potato, white-fleshed sweet potato, butternut squash, parsnip, sweetcorn, carrot, peas, tomato paste, tomato purée, onion	Raw dehydrated starchy vegetables (carrot, onion, peas, potato)
Fruit		Grapefruit, melon, youngberry	Canned fruit, stewed fruit, raw fruit	Dried fruit
Legumes and legume products		Raw tofu, cooked soybeans	Beans, rice and lentil dishes, lentils, raw soybeans, cooked chickpeas	Dried beans, dried chickpeas
Nuts and seeds	Sesame seeds	Coconut, pecan nuts	Nuts and seeds	Chestnuts
Milk and milk products	Some cheeses (medium/reduced fat, Leicester, Gouda)	Cheese, sour milk	Milk, yoghurt, custard	Skim and low-fat milk powders
Eggs	Raw chicken egg (omega-3 enriched), raw quail egg	Eggs	Soufflé	
Meat and meat products	Offal, mutton, beef heart, beef kidney, beef patty	Frankfurter, pastrami, offal, luncheon meat, bacon, sausage, meatball, schnitzel, liver, ham, steak and kidney, chicken giblets	Commercial meat pies, meat spread, biltong, pâté, stews with meat and vegetables, corned beef	
Fish and seafood	Baked/fried fish	Boiled shrimp/prawn, baked kipper, oyster, caviar	Battered/crumbed fish, mussel, rollmop/pickled herring	
Fats and oils	Butter ghee, margarine	Cream	Salad dressing, peanut butter	
Sugar, syrups and sweets				Sugar and sweets
Soups, sauces, seasonings and flavorings		Cucumber soup, meat gravy, snakehead soup	Sauces	Caramel sauce
Beverages	Coffee, tea		Fruit juices, milk beverages	Malted milk powder, drinking chocolate powder
Infant and paediatric feeds and foods			Reconstituted infant feeds	Infant feed powders
Therapeutic/special/diet products			Reconstituted therapeutic products	Therapeutic powders
Miscellaneous		Wine	Baking powder, liqueur with cream, sherry	Liqueur

**Table 5:** Food items within the identified fatty acid classes.

Class	Class 1	Class 2
Class description	Low content	High content
Food group		
Cereals and cereal products	Maize, wheat, barley	Baked goods
Vegetables	All vegetables	
Fruit	All fruit	
Legumes and legume products	Beans, lentils	
Nuts and seeds		Nuts and seeds
Milk and milk products	Skim milk, fat-free cottage cheese	Other milk and milk products
Eggs		Eggs
Meat and meat products		All meat and meat products
Fish and seafood	Tuna, crab, haddock, low-fat fish	Caviar, high-fat fish
Fats and oils		Fats and oils, fried foods
Sugar, syrups and sweets	Molasses	Chocolate, icing
Soups, sauces, seasonings and flavorings		Sauces
Beverages		Milk beverages
Infant and paediatric feeds and foods		Infant feeds
Therapeutic/special/diet products		Some therapeutic powders

**Table 6:** Food items within the identified iron classes.

Class	Class 1	Class 2	Class 3
Class description	Low content	High content	Moderate content
Food group			
Cereals and cereal products	Super/special soft maize meal porridge (unfortified), low-fat milk and whole milk pudding (blancmange, instant)	Wheat flour, oats, semolina, baked goods, pasta dishes, raw maize meal, bread	Stiff and crumbly maize meal porridge, fortified soft maize meal porridge, rice, rice flour
Vegetables	Squash, tomato, asparagus	Leaves, dehydrated raw vegetables, peas, spinach, broccoli, Brussels sprouts, green beans, beetroot, baby marrow squash, mushroom, tomato juice	Brinjal, cabbage, sweetcorn, squash, sweet potato, tomato, onion, potato, parsnip, cauliflower, carrots
Fruit	Apple, lemon juice, grapefruit, naartjie, pawpaw, watermelon, cherries, nectarine, canned peaches, canned pears, rhubarb	Dried fruit, prune juice	Apricot, avocado, guava, canned fruit, figs, pears, prunes, granadilla, dates, grapes, peaches, plums, pineapple, fruits nectars (apricot, pear), fruit juices (grapefruit, pineapple, grape)
Legumes and legume products		Legumes and legume products	
Nuts and seeds		Nuts and seeds	
Milk and milk products	Milk, yoghurt, custard, reconstituted skim milk powder	Milk powder with added iron, cheese (feta, cottage, Gouda)	Milk powders, evaporated milk, custard
Eggs		Eggs	
Meat and meat products		Meat and meat products	Chicken (white meat), veal, chicken stew
Fish and seafood		Anchovy, oyster, sardines, mussels, tuna, fried fish, shrimp/prawn	Low-fat fish, shrimp/prawn, crab, salmon, sole
Fats and oils	Vegetable oil, cream, French salad dressing, butter ghee, butter and hard margarine (mixed), coconut oil, soybean oil	Peanut butter, canned cream	Olive oil, salad dressing
Sugar, syrups and sweets	Icing, sugar	Chocolate, jam/marmalade, molasses	Honey
Soups, sauces, seasonings and flavorings		Curry sauce, soups with meat and vegetables	Sauces
Beverages	Malted milk beverages, coffee, tea	Malted milk powder, drinking chocolate powder	Malted milk beverages, drinking chocolate powder
Infant and paediatric feeds and foods		Infant feeds	
Therapeutic/special/diet products		Therapeutic powders	
Miscellaneous	Spirits, liqueur, vinegar	Baking powder	Wine, sherry

The study found that the classification of food items using moisture (Supplementary Table 1), animal protein (Table 7) and sodium (Table 3) content could be described by low, moderately-low, moderately-high and high nutrient content classes. Based on the saturated, mono-unsaturated and polyunsaturated fatty acid content, food items could be described by low- and high-content classes (Table 5). When examining their available carbohydrate content, food items could be described as having an extremely low, low, moderate and high available carbohydrate content (Table 4). Low, moderate and high nutrient content classes of food items were also identified based on vitamin A (RE) and thiamin content (Supplementary Table 3). While most food items exhibited a clear belonging to classes, a few food items exhibited multiclass membership. For example, for vitamin A (RE) content, raw leaves other than amaranth had an approximately equal probability of belonging to either the moderate content or high content class while amaranth leaves had a clear belonging to the high content class. Other classes of interest are shown in Supplementary Tables 2–5. Food items with a high copper content were identified by class 2 and consisted of wheat flour, maize meal, leafy greens, mushrooms, potatoes, beans, lentils, nuts and seeds, organ meat, shellfish and chocolate (Supplementary Table 2). The distributions for cholesterol and manganese did not display classes that could be intuitively ranked (Supplementary Table 6).

**Table 7:** Food items within the identified animal protein classes.

Class	Class 1	Class 2	Class 3	Class 4
Class description	Low content	Moderately-low content	Moderately-high content	High content
Food group				
Cereals and cereal products	Rice cooked with margarine, pastry/crust made with margarine	Puddings, baked goods, pasta dishes	Tuna pie	
Vegetables		Vegetables coated in batter		
Legumes and legume products	Beans cooked with margarine	Lentils with egg		
Milk and milk products		Milk, yoghurt	Cottage cheese, feta	Milk powder, cheese
Eggs		Scrambled egg, soufflé	Raw egg, fried egg	
Meat and meat products		Commercial meat pies, pork/beef sandwich spread, ham and tongue loaf, offal	Chicken, ham, meat stews/curries, duck, Frankfurters, sausage, pâté, luncheon meat, corned beef	Beef, pork, veal, mutton, turkey, goose, pork sausage, salami
Fish and seafood		Fish biltong, oyster, low-fat fish cakes, fish fingers	Crab, fatty fish, baked/crumbed fish, sole, rollmop/pickled herring	Anchovy, tuna, fish, haddock, sardines, caviar, kipper, mussels, salmon, shrimp/prawn
Fats and oils	Butter ghee	Cream, butter, margarine, homemade salad dressing		
Sugar, syrups and sweets	Icing, dark chocolate	Chocolate, jelly, cottage cheese icing		
Soups, sauces, seasonings and flavorings		Sauces, soups with beef		
Beverages		Milk beverages, eggnog		
Infant and paediatric feeds and foods		Reconstituted infant feeds	Whey-predominant infant feed powder	
Therapeutic/special/diet products		Reconstituted therapeutic products		Some therapeutic powders
Miscellaneous		Liqueur with cream		

Classes capturing foods exhibiting extreme values of nutrients were also identified. These classes contained both foods having low or extremely low nutrient content and foods having a high or extremely high nutrient content. This class was present in the distributions for magnesium, potassium, sodium, copper, riboflavin and pantothenic acid. The distribution of phosphorous also contained two classes with class 1 describing foods with extremely low phosphorous content such as marrow squash, tomato juice, butter ghee, margarine, tea and baking powder.

### 3.3. Class validity

**Figure 4:**
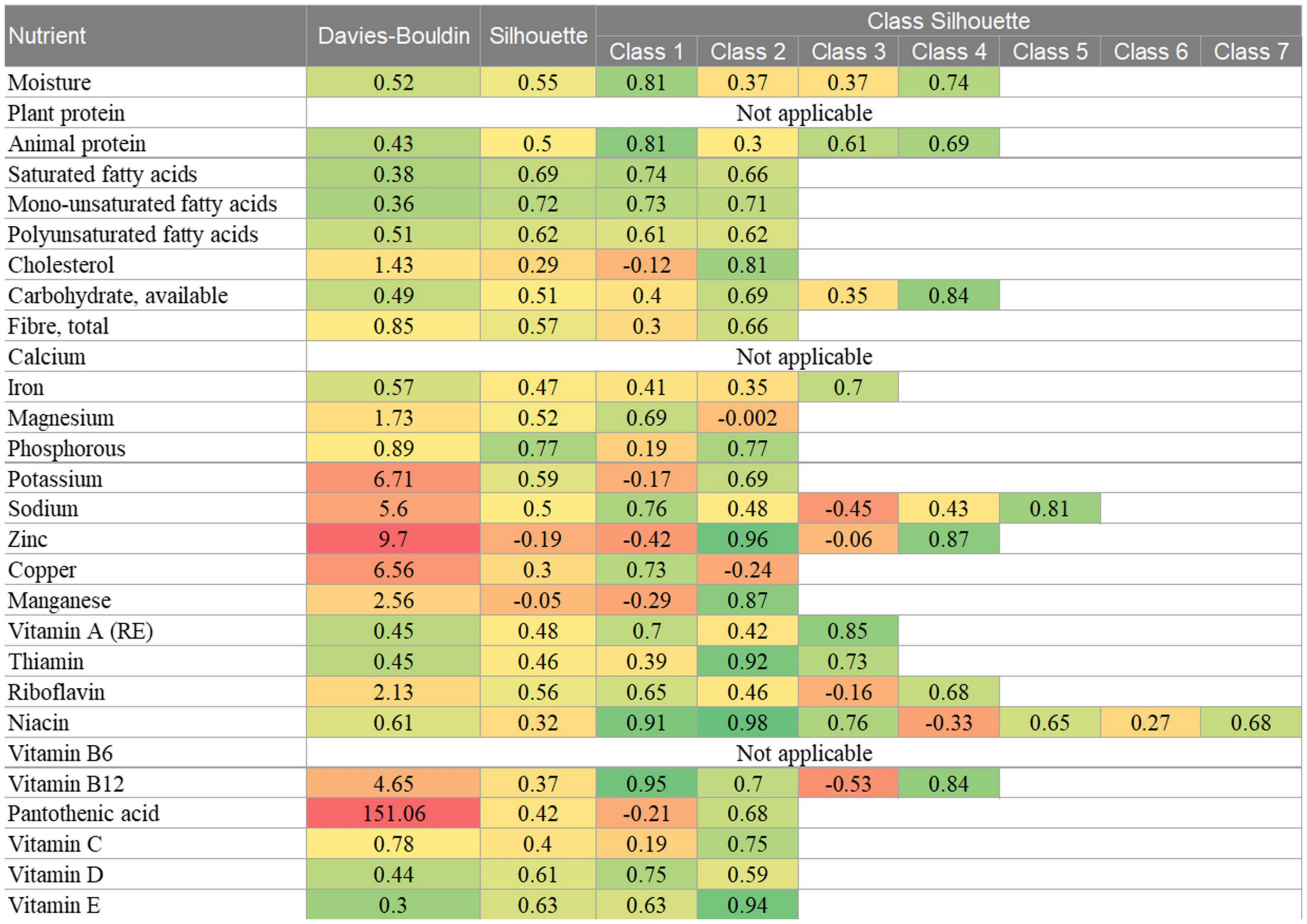
Internal class validity indices for the univariate GMM classifications. Key: green = good score, yellow = moderate score, red = poor score.

The internal class validity indices are presented in Figure 4. Nutrient-based classifications with good DB indices and silhouette coefficients are indicated by green shading. The median DB index was 0.78 (IQR 0.45–4.08), suggesting that the GMM resulted in good classification. The minimum score was 0.3 for the vitamin E classifications and the highest scores, ranging from 4.65 to 9.7, were found for the potassium, sodium, zinc, copper and vitamin B_12_ classifications. An outlying score of 151.06 was found for the classification by pantothenic acid. Each of the classifications that were found to have a high DB score, contained a class that simultaneously captured foods with extremely high nutrient levels and foods with extremely low nutrient levels. For example, class 2 of the copper classification accounted for 56% of the food items and contained foods with both extremely low and extremely high levels of copper. Thus, classifications that contained such a class, tended to have the most overlap of classes and were congruent with having high DB scores.

The median silhouette coefficient was 0.5 (IQR 0.39–0.62), also suggesting that the GMM resulted in good classification. Negative silhouette coefficients were found for the zinc and manganese classifications, both of which had a significant overlap of classes. When examining the coefficients for each class, both the zinc and manganese classifications had some classes with high coefficients, suggesting that observations within these classes displayed good cohesion. Again, individual class coefficients were low for classes that captured both extremely high and extremely low values. For example, the first class of cholesterol accounted for 65% of the food items and described foods with either an extremely low cholesterol value or an extremely high cholesterol value. This class had a silhouette coefficient of −0.12 compared with the second cholesterol class which scored 0.81. This similar pattern was also seen for the potassium, sodium, zinc, copper, manganese, riboflavin, niacin and vitamin B_12_ classifications. Classes that had a significant overlap with other classes tended to have a negative silhouette coefficient.

## 4. Discussion

In this paper, we have applied Gaussian mixture models to the South African Food Composition Database to evaluate the application of probabilistic classification to food composition data. The classification of food items into nutritionally similar food groups is a common objective of studies that apply statistical methods for the analysis food composition data. Traditional food groupings are not enough to describe the nutritional landscape of food and compositionally similar food groups also need to be investigated. Identifying compositionally similar food groups can be achieved through clustering algorithms which are simple to employ. However, most of the clustering algorithms applied thus far assign food items exclusively to one class and the indistinct thresholds that may exist between food groups, based on nutritional content, needs to be considered. The application of probabilistic clustering can account for this uncertainty.

An important application of FCDBs is its role in the design of therapeutic diets (23). Renal disease, diabetes mellitus and anaemia are some examples of health conditions that require the monitoring of specific nutrients. Allowed food lists and food exchange lists are a useful tool for health practitioners and patients when managing such conditions. They are also useful for healthy individuals to improve their nutrition education (24). Applying clustering methods to food composition data provides a data-driven method of establishing foods with similar nutritional content, for the development of allowed food lists.

Classifications based on cholesterol, total fibre, magnesium, potassium, copper and pantothenic acid content, indicated a clear overlap of two classes, supporting the use of probabilistic classification methods. The differing class variances also suggest that the *k*-means clustering algorithm may be less suitable when applied to food items since the *k*-means algorithm separates items into groups of equal variance.

The classes obtained from the GMMs provided greater detail when compared to the groupings identified in a previous study that applied principal component analysis to the SAFCDB to identify compositionally similar food items (7). While the PCA groupings identified the ‘meat and meat products’ food category as a whole being high in animal protein, the identified GMM classes based on animal protein was able to further separate this food category into three subclasses. Specific food items, such as red meat and oily/fatty fish were identified to be high in animal protein. Similarly, while the PCA groupings identified leaves such as lambs quarters and sow thistle leaves as containing a high vitamin A content, our analysis has shown that only amaranth leaves exhibit a higher than average vitamin A content. Thus, within broad food categories, our classification provides detailed subcategories with a focus on the individual food items. In addition, regarding the vitamin A content of leaves other than amaranth leaves, other leaves had an approximately equal probability of belonging to either the moderate content class or the high content class. This finding emphasizes the uncertain thresholds between clusters in food composition data and is possible to quantify through evaluating the class membership probabilities, available with probabilistic classification and is an advantage over PCA.

Although there was a discernible link between the identified classes and the SAFCDB food groups, the identified classes included food items from various SAFCDB food groups. This suggests that compositional similarity cannot be completely described by traditional food groups such as grains, vegetables and dairy, which was a similar finding in other studies (25–27). This also supports the nutritional practice of disease specific food exchange lists in diet therapy, such as renal exchange lists, that are informed by the nutrients of concern. Individuals with kidney disease are advised to follow the renal diet (8) which limits particular nutrients, such as protein, sodium, phosphate and potassium. Our analysis classified food items such as rice, pasta, marrow and peach and pear nectars as low potassium foods. Food items such as potatoes, dried raw vegetables, some nuts, milk powder, fish biltong and molasses were found to have a high potassium content. This is consistent with the recommended list of foods to consume and avoid when controlling potassium intake according to the renal diet (28).

Limiting sodium is also necessary for both kidney disease and hypertension (29). Foods identified as having the highest sodium content were bread, potato crisps, canned vegetables, processed meat and instant soups which is consistent with the recommended foods to avoid (30). Foods with the lowest sodium content were mostly fruit and vegetables with some fruit and vegetables containing less sodium than others, an aspect which was easily identifiable from our results and consistent with the recommendations of the DASH diet (31). Since this is data from before the current salt regulations (32) were implemented, future work could explore the impact of the salt regulations on the sodium content of foods using an updated version of the SAFCDB.

Carbohydrate content is also often monitored as part of a healthy diet to control type 2 diabetes and metabolic syndrome (33). Foods identified in the high available carbohydrate class, such as baked goods, starchy vegetables and sweets, are often considered as a source of low-quality carbohydrates (34, 35) and individuals can use this ranking as a guide on foods to monitor when following a low-carbohydrate diet. The foods found in our carbohydrate classes align with the classification of foods by GI (36). Low GI foods such as non-starchy vegetables, fruit and protein-rich foods were grouped together as foods with a low carbohydrate content. In addition, milling was a common processing method in the high available carbohydrate content group and this is known to increase the glycaemic index (GI) of certain foods (finer food particles increase absorption contributing to a higher GI) (29). Using our results, similar food lists can be developed for anaemia and hemochromatosis (requires the control of iron intake), Wilson’s disease (requires the control of copper intake), coronary heart disease (requires the control of fatty acid and dietary cholesterol intake), and gut health (impacted by total fibre intake). Using GMM to classify food items for the development of food lists provides objective rankings of food items while also accounting for the structure of food composition data. Since GMM is a data-driven method, the process of ranking food items using this method reduces the need for manual categorization and food groups can easily be reassessed with the addition of more or updated data.

Food composition data has similar methodological challenges to that of food consumption data such as right-skewness and a large proportion of food items having zero content of a particular nutrient (37). Using a log-transform before applying the GMM adjusted for the skewness and enabled the patterns of each nutrient distribution to become discernible. This also revealed that the distribution of nutrients could be modeled as mixture of Gaussians and foods with a zero nutrient content could be easily excluded from the univariate analysis. This is a desirable property since we are only interested in classifying foods known to have a particular nutrient. The separation of zero nutrient content foods from foods known to have the nutrient was also advocated for by Khan (4). In addition, classes capturing food items with either an extremely low nutrient content or an extremely high nutrient content were also identified. This facilitates outlier detection which could represent foods with an actual extreme nutrient content, foods with added components such as added sugar or added salt, or foods with erroneous values for a specific nutrient. Using extreme values to identify errors was also previously investigated (38).

Overall, the class validity indices indicated that application of the GMM resulted in good classification. Classes with a substantial overlap between them were shown to have poorer internal validity scores than classes that were more separable. Since internal indices focus on separability as one of the criteria for class validity, these indices are unsuitable when the data displays mixed class membership. Further research is needed on appropriate internal class validity indices in the presence of overlapping classes obtained through GMM clustering and on the stability of the identified classes.

The univariate GMM provided useful results but multiple nutrients are present in food items and thus multiple nutrients are consumed simultaneously. While it is important to know which foods may have a relatively low or high nutrient content, consuming a food high in particular nutrient may also unknowingly increase the intake of other nutrients. Thus, it is important to consider the multivariate GMM as future work. However, this can be challenging in the case of high-dimensional data such as food composition data. GMMs often fit extra classes to capture the outliers and can result in poor data fit. Future work could investigate the mixture of multivariate t-distributions (39) to account for the long tails and outliers seen in our data and incorporating the structural zeroes into the clustering algorithm using a zero-inflation model could also be explored (40). Alternatively, Lo and Gottardo (41) proposed a multivariate t-distribution with Box-Cox transformation that could simultaneously address data transformation and outlier detection which are characteristics pertinent to the analysis food composition data.

In conclusion, this study has explored the application of univariate Gaussian mixture models to examine the classification of food items within the South African Food Composition Database. The identified classes exhibited overlap, supporting the use of probabilistic classification methods to account for the uncertainty of nutrient thresholds between classes. Classifying food items by moisture, animal protein, fatty acid, available carbohydrate, total fibre, sodium, vitamin A, thiamin and riboflavin content produced classes with differing means and estimates of variability and could be clearly ranked on a low to high nutrient content scale. Our results highlight that classifications within the broader, traditional food groups exist and our method focuses on identifying the individual food items within these subclasses. The results can be used to inform the development of nutrient profiling indices, allowed food lists and food-based dietary guidelines. The identified classes could also be incorporated into food composition databases to provide an additional level of classification and understanding of food items, thus promoting nutrition education for the user. Since we included processed and manufactured food items in our analysis, manufacturers can use these findings to inform product formulation as well.

## Data availability statement

The original contributions presented in the study are included in the article/Supplementary material, further inquiries can be directed to the corresponding author.

## Author contributions

YB and SM contributed to the conception and design of the study. AG provided access to the database. YB performed the statistical analysis and wrote the first draft of the manuscript. SM, HM, and AG provided supervision. All authors contributed to manuscript revision and approved the submitted version.
