# Supplementary material for: Determining classes of food items for health requirements and nutrition guidelines using Gaussian mixture models
Source: Front Nutr. 2023 Oct 13;10:1186221. doi: 10.3389/fnut.2023.1186221 (PMC10611470; doi:10.3389/fnut.2023.1186221)
Supplement: Supplementary file 1 [file Data_Sheet_1.docx]

Supplementary Material

Determining classes of food items for health requirements and nutrition guidelines using Gaussian mixture models

**Yusentha Balakrishna*, Samuel Manda, Henry Mwambi, Averalda van Graan**

**Correspondence:** Yusentha Balakrishna: yusentha.balakrishna@mrc.ac.za

# Supplementary Tables

Supplementary Table 1. Food items in classes of interest for moisture.

| Class | Class 1 | Class 2 | Class 3 | Class 4 |
| --- | --- | --- | --- | --- |
| Class description | Low content | Moderately-low content | Moderately-high content | High content |
| Food group |  |  |  |  |
| Cereals and cereal products | Breakfast cereals, rusks, oats, wheat germ, pastry/crust, cookies, raw maize meal, flours | Cakes, bread, puddings, tarts/pies, crumbly maize meal porridge | Cooked pasta, stiff maize meal porridge, cooked barley, cooked rice, milk tarts, apple pie | Cooked oats, wheat porridge, soft maize meal porridge |
| Vegetables |  |  | Potato, peas, sweetcorn, parsnip, squash, brinjal, onion | Vegetables |
| Fruit | Dehydrated raw vegetables, dried fruit rolls, raisins | Fruit mincemeat, dried fruit | Stewed fruit, canned fruit | Fruit |
| Legumes and legume products | Dried legumes |  | Cooked legumes | Raw tofu |
| Nuts and seeds | Nuts and seeds | Chestnuts, coconut |  |  |
| Milk and milk products | Milk powders | Cheese | Evaporated milk, ice cream | Milk, cottage cheese, yoghurt |
| Eggs | Dried egg | Raw egg yolk | Eggs |  |
| Meat and meat products | Cured bacon | Commercial meat pies, biltong, pepperoni, salami, sausage, chicken skin | Meat and meat products | Raw beef tripe |
| Fish and seafood |  | Caviar | Fish and seafood | Oyster, sole |
| Fats and oils | Peanut butter, butter ghee | Mayonnaise, margarine, butter | Homemade salad dressing, cream |  |
| Sugar, syrups and sweets | Icing, chocolate | Jam/marmalade, molasses, honey | Cottage cheese icing, savoury sauces | Jelly |
| Soups, sauces, seasonings and flavorings |  | Chocolate sauce, caramel sauce |  |  |
| Beverages | Malted milk powder, drinking chocolate powder |  | Milkshake | Tea, coffee |
| Infant and paediatric feeds and foods | Infant feed powders |  |  | Reconstituted infant feeds |
| Therapeutic/special/diet products | Therapeutic powders |  | Reconstituted therapeutic products |  |
| Miscellaneous | Baking powder | Liqueur | Sherry, spirits | Vinegar, wine and water |

Supplementary Table 2. Food items in classes of interest for total fibre, magnesium, potassium, zinc and copper content.

| Nutrient | Fibre, total | Magnesium | | Potassium | | Zinc | | Copper | |
| --- | --- | --- | --- | --- | --- | --- | --- | --- | --- |
| Class | Class 2 | Class 2 | | Class 1 | | Class 4 | Class 3 | Class 2 | |
| Class description | High content | Extremely low content | Extremely high content | Extremely low content | Extremely high content | High content | Extremely high content | Low content | High content |
| Food group |  |  | |  | |  |  |  | |
| Cereals and cereal products | Bread, maize meal, pasta dishes, rice, scones, roti |  | Wheat germ, breakfast cereals, oats, wheat flour, bread, wholewheat baked goods, potato crisps | Rice, pasta, soft maize meal, pastry, pudding | Were potato flour and potato crisps | Fortified brown bread, oats, fortified maize meal, muesli | Wheat germ, puffed corn cereal | Tarts/pies, puddings, wheat products, soft maize meal | Breakfast cereals, wheat germ, wheat flour, rye, oats, bread, raw maize meal, oat cookies, fruit cake |
| Vegetables | All vegetables excluding dehydrated vegetables | Marrow squash | Leaves, dehydrated raw vegetables, spinach, tomato paste, beetroot, okra, broccoli | Marrow squash | Potato, dehydrated raw vegetables, tomato paste | Dehydrated peas, dehydrated cauliflower, dried amaranth leaves |  | Cabbage, carrot, sweetcorn, celery, cucumber, rhubarb | Dehydrated raw vegetables, leaves (amaranth, cowpea, etc.), mushroom, potato, tomato paste/purée, green pepper, squash, brinjal, asparagus, peas |
| Fruit | All fruit excluding fruit juices | Apple, blueberry, olives | Dried fruit, raisins, currants, prunes |  | Dried fruit | Figs |  | Apple, lemon, naartjie, prune | Avocado, currants, plums, apricots, raisins, pear, peach, fig |
| Legumes and legume products | All cooked beans and lentils |  | Dried legumes |  | Dried raw beans and lentils | Dried raw legumes |  |  | Soybeans, beans, lentils, chickpeas, tofu |
| Nuts and seeds | All nuts and seeds |  | Nuts and seeds |  | Peanuts, almonds, pistachios | Pine nuts, almonds, peanuts, cashew nuts, sunflower seeds, sesame seeds, brazil nuts, pecan nuts, walnuts |  |  | Nuts and seeds |
| Milk and milk products |  |  | Milk powders |  | Milk powder (goat and added vitamins) | Fortified milk powder, cheese (Cheddar, Gouda, Leicester, fortified) |  | Milk and milk powders, cottage cheese, yoghurt, custard |  |
| Eggs |  |  | Dried egg |  |  | Dried whole chicken egg, raw chicken egg yolk |  | Dried egg and raw yolk (chicken), soufflé |  |
| Meat and meat products | Meat dishes with vegetables (e.g. Stew) |  | Beef biltong, cured bacon | Trotters |  | Beef, organ meat, veal, mutton, chicken giblets | Beef chuck, beef biltong | Chicken (white meat) | Organ meat, veal, goose, duck |
| Fish and seafood |  |  | Fish biltong, anchovy, haddock, oyster, medium and high-fat fish, shrimp/prawn, sardine |  | Fish biltong | Crab | Oyster | Low fat fish | Shellfish, anchovy |
| Fats and oils |  | Butter ghee, margarine, mayonnaise | Peanut butter | Butter, margarine, olive oil |  |  |  | Fats and oils |  |
| Sugar, syrups and sweets | Chocolate coated nuts and raisins, chocolate icing, jam/marmalade | Icing, honey | Chocolate, molasses | Sugar, icing, honey | Molasses |  |  |  | Chocolate |
| Soups, sauces, seasonings and flavorings | Soups and sauces with vegetables |  |  | Savoury sauces |  |  |  | White sauces, cheese sauces |  |
| Beverages | Drinking chocolate | Fruit nectars | Drinking chocolate powder | Pear and peach nectar |  |  |  | Malted milk beverages, fruit juice | Drinking chocolate |
| Infant and paediatric feeds and foods |  |  | Infant powders |  |  | Some infant feeds |  |  | Some infant feeds |
| Therapeutic/special/diet products |  | Some therapeutic products | Some therapeutic powders |  | Some therapeutic powders | Some therapeutic powders |  |  | Therapeutic powders |
| Miscellaneous |  | Tea, coffee, vinegar, liqueur | Baking powder | Tea, vinegar, baking powder, spirit/brandy, liqueur |  |  |  | Coffee, tea, wine, vinegar |  |

Supplementary Table 3. Food items in classes of interest for vitamin A (RE), thiamin and riboflavin content.

| Nutrient | Vitamin A (RE) | | Thiamin | | Riboflavin | | | |
| --- | --- | --- | --- | --- | --- | --- | --- | --- |
| Class | Class 1 | Class 3 | Class 2 | Class 3 | Class 3 | Class 1 | Class 4 | Class 3 |
| Class description | Low content | High content | Low content | High content | Extremely low content | Low content | High content | Extremely high content |
| Food group |  |  |  |  |  |  |  |  |
| Cereals and cereal products | Crushed wheat, wheat flour, raw maize grit, raw maize meal, unfortified super maize meal, wholewheat pasta, barley, oats |  |  | Wheat germ, wheat flour, raw maize meal, whole kernel raw maize, uncooked oats, breakfast cereals, baked goods, bread, cooked pasta |  | Rice, rice flour, cooked oats, maize flour, crushed wheat, soft maize meal, raw maize grit | Baked goods, pasta dishes, wholewheat flour, uncooked oats, fortified raw maize meal | Breakfast cereals, wheat germ, wheat flour, semolina |
| Vegetables | Potato, sou sou, white-fleshed sweet potato, gem squash, cauliflower, cabbage | Carrots, orange-fleshed sweet potato, dried raw amaranth leaves | Olives, boiled brinjal, boiled amaranth leaves | Dehydrated raw vegetables, peas, cowpea leaves |  | Vegetables cooked with margarine, cabbage, cauliflower, squash, potato, brinjal, cucumber, celery, tomato, sweet potato, peas, lettuce, beetroot, sou sou, pumpkin, onion, carrots, green pepper | Mushroom, leaves | Dried amaranth leaves, dehydrated raw green beans, dehydrated raw cauliflower |
| Fruit | Sultanas, grapefruit, guava, pear, figs, lemon, apple, raisins, grapes, avocado, dates, pineapple, raspberry, blueberry, youngberry, cherries, melon, mineola, orange, strawberry, pear nectar, fruit juices (lemon, prune, pineapple, grapefruit, grape) | Dried apricot roll | Peaches, pears, cherries, granadilla, litchis |  | Starking apple, glazed cherries | Raw fruit, canned fruit, fruit juices | Certain dried fruit (peaches, pears, apple, currants, prunes), dried apricot roll |  |
| Legumes and legume products | Cooked legumes, tofu |  |  | Raw legumes |  |  | Raw legumes | Dried soybeans |
| Nuts and seeds | Almonds, walnuts, hazelnuts, chestnuts, pine nuts |  |  | Dried nuts and seeds |  | Coconut | Dried nuts and seeds, cashew nuts, chestnuts | Almonds |
| Milk and milk products | Drinking yoghurt, fat-free cottage cheese |  |  | Milk powders (added vitamins, goat) |  |  | All other milk and milk products | Milk powders (added vitamins, goat), cheese (leicester, derby) |
| Eggs |  |  |  | Dried egg, raw egg yolk |  |  | Cooked eggs | Whole eggs, egg yolk |
| Meat and meat products | Pork | Liver, giblets |  | Pork, beef |  |  | Beef, mutton, pork, veal, turkey | Organ meat |
| Fish and seafood | Crab |  |  | Fish biltong |  | Shrimp/prawn, sole | Medium- and high-fat fish, salmon, sardines, anchovy, canned oyster | Caviar |
| Fats and oils |  |  |  |  | Butter ghee, French salad dressing | Mayonnaise, margarine (polyunsaturated) | Cream, peanut butter |  |
| Sugar, syrups and sweets |  |  | Jam/marmalade |  | Molasses, jam/marmalade | Sugar, icing, coconut ice | Chocolate |  |
| Soups, sauces, seasonings and flavorings |  |  |  | Soup packet mix |  | Curry sauce, cucumber soup | White sauce, cheese sauce |  |
| Beverages |  |  |  | Malted milk powder | Herbal tea, spirits | Reconstituted drinking chocolate, tea, alcohol | Milk beverages, eggnog | Malted milk powder |
| Infant and paediatric feeds and foods |  |  |  | Infant feed powders |  |  |  | Infant feed powders |
| Therapeutic/special/diet products |  |  |  | Therapeutic powders |  |  |  | Therapeutic powders |

Supplementary Table 4. Food items in classes of interest for niacin and vitamin B_12_ content.

| Nutrient | Niacin | | | | | Vitamin B_12_ | |
| --- | --- | --- | --- | --- | --- | --- | --- |
| Class | Class 1 | Class 2 | Class 6 | Class 5 | Class 7 | Class 1 | Class 3 |
| Class description | Extremely low content | Low content | Moderately-high content | High content | Extremely high content | Extremely low content | Extremely high content |
| Food group |  |  |  |  |  |  |  |
| Cereals and cereal products | Canned apple pie | Cooked oats | Bread, cakes, cooked barley, cookies, pasta dishes, raw maize meal, maize meal porridge, whole kernel maize, uncooked oats, pastries, pudding, potato flour, white rice flour, cooked rice, baked goods, brown wheat flour | Brown rice flour, fortified bread/rolls, uncooked semolina, wheat flour, wheat germ, fortified maize meal | Breakfast cereals | Cooked white rice, fruit cake, apple pudding |  |
| Vegetables |  | Frozen brinjal, English cucumber, canned baby sweetcorn, onion, canned sauerkraut | Asparagus, dark green leaves, sweetcorn, peas, potato, squash, tomato | Mushroom, dehydrated potato, dried amaranth leaves, dehydrated cauliflower, dehydrated green beans |  | Vegetables cooked with margarine |  |
| Fruit | Glazed cherry, canned litchi | Apple (peeled, baked, stewed), canned sultanas, naartjie juice | Apricot, prune, peach, avocado, gooseberry, guava, plum, date, fig, granadilla, raisins, dried fruit | Dried peach |  |  |  |
| Legumes and legume products |  | Fried tofu | Dried raw legumes and lentils |  |  |  |  |
| Nuts and seeds |  |  | Almonds, macadamia, hazelnut, cashew nuts, chestnut, pistachio, coconut, pecan nut, walnut | Sesame seeds, sunflower seeds, pine nuts | Peanuts, peanut butter |  |  |
| Milk and milk products | Derby cheese, reduced fat cheese | Cheese, reconstituted milk powder, milk, yoghurt | Feta cheese, fortified milk powder, goat milk powder |  |  |  | Fortified milk powder |
| Eggs | Raw chicken egg yolk | Whole raw chicken egg, fried/scrambled/poached eggs |  |  |  |  | Whole raw egg |
| Meat and meat products |  |  | Processed meat, dark chicken meat, offal | Beef, chicken, duck, goose, mutton, pork, veal (dry) | Liver, chicken (white meat), turkey, bacon |  | Organ meat, chicken giblets |
| Fish and seafood |  |  | Low-fat fish, shrimp/prawn, crab, oyster | Salmon, sardine, medium- and high-fat fish | Anchovy, tuna |  | Caviar, oyster, mussel, sardine, salmon, crab and medium/high-fat fish |
| Fats and oils | Butter ghee, margarine, mayonnaise | Canned cream |  |  |  | Butter ghee |  |
| Sugar, syrups and sweets | Jam/marmalade |  | Molasses |  |  |  |  |
| Soups, sauces, seasonings and flavorings |  |  |  | Soup mix |  |  |  |
| Beverages |  |  |  | Malted milk powder |  |  |  |
| Infant and paediatric feeds and foods |  |  | Some infant feeds | Some infant feeds |  |  |  |
| Therapeutic/special/diet products |  |  | Some therapeutic powders |  | Some therapeutic powders |  |  |

Supplementary Table 5. Food items in classes of interest for pantothenic acid, vitamin D and vitamin E content.

| Nutrient | Pantothenic acid | | Vitamin C | Vitamin D | Vitamin E |
| --- | --- | --- | --- | --- | --- |
| Class | Class 1 | | Class 2 | Class 1 | Class 2 |
| Class description | Extremely low content | Extremely high content | High content* | Extremely low content | Extremely low content |
| Food group |  | |  |  |  |
| Cereals and cereal products | Puddings, pastry crusts, tarts, soft maize meal | Rice flour, wheat germ, oats, dehydrated and raw vegetables (mushroom, broccoli, cauliflower, cabbage, onion, potato, sweet potato, peas and truffles), dried amaranth leaves, avocado | Apple pudding/tarts, fried rice, potato crisps, puffed corn cereal | Puddings | Beetroot, marrow squash, green beans, truffles |
| Vegetables | Boiled leaves (amaranth, cowpea, etc.), frozen/boiled vegetables |  | Cabbage, potato, sweet potato, tomato, dark leafy greens, broccoli, brussels sprouts, parsnip, squash, sweetcorn, green beans, peas, onion, asparagus, cucumber | Okra, cauliflower, potato | Cherries, prunes |
| Fruit | Canned/stewed fruit |  | Most raw fruits, citrus fruit juices |  |  |
| Legumes and legume products |  | Raw beans, lentils, split peas, chickpeas and soybeans |  |  |  |
| Nuts and seeds |  | Raw/roasted peanuts, cashew nuts, sunflower seeds and hazelnuts | Chestnut |  | Drinking yoghurt, fat-free cottage cheese, reconstituted skim milk powder |
| Milk and milk products |  | Milk powder (fortified and goat), feta cheese | Fortified milk powder, goat milk powder | Milk, cottage cheese, yoghurt, custard (made with milk and custard powder) |  |
| Eggs |  | All eggs |  |  |  |
| Meat and meat products |  | Organ meat, poultry, beef, pork |  | Chicken without skin |  |
| Fish and seafood |  | Caviar, crab, medium- and high-fat fish |  |  |  |
| Fats and oils | Butter, margarine | Peanut butter |  | Canned cream |  |
| Sugar, syrups and sweets | Icing | Candied orange/lemon peel | Jam/marmalade |  |  |
| Soups, sauces, seasonings and flavorings | Curry sauces, gravy |  |  | Soups |  |
| Beverages |  |  |  | Malted milk beverage |  |
| Infant and paediatric feeds and foods |  | Some infant feeds | Some infant feeds |  |  |
| Therapeutic/special/diet products |  | All therapeutic powders | Some therapeutic powders |  |  |
| Miscellaneous |  | Wine, sherry, tea, liqueur |  |  |  |

* Foods that were associated with class 1 but which were also identified to be high in vitamin C were guava, marula, kiwifruit, pawpaw, lemon, plum, strawberry, cabbage, cauliflower, broccoli, green pepper and mangetout.

Supplementary Table 6. Food items in classes for cholesterol and manganese.

| Nutrient | Cholesterol | Manganese |
| --- | --- | --- |
| Class | Class 2 | Class 2 |
| Class description | None | None |
| Food group |  |  |
| Cereals and cereal products | Baked goods | Pasta dishes, cakes, maize flour, maize meal, pastry, pudding, tarts/pies |
| Vegetables |  | Brinjal, broccoli, cabbage, carrot, cauliflower, cucumber, green beans, sweetcorn, mushroom, onion, potato, baby marrow, lettuce, celery |
| Fruit |  | Guava, berries, mango, apricot, prune, raisins, fig, dried fruit, prune juice |
| Legumes and legume products |  |  |
| Nuts and seeds |  |  |
| Milk and milk products | Custard, processed cheese |  |
| Eggs | Soufflé | Chicken egg (whole, dried), raw chicken yolk |
| Meat and meat products | Beef, pork, chicken (dark meat, skin), sausages | Chicken giblets, beef kidney, chicken liver pâté |
| Fish and seafood | Fried fish, salmon, crab, canned oyster |  |
| Fats and oils | Canned and pressurized cream, butter and hard margarine, cooked salad dressing |  |
| Sugar, syrups and sweets |  |  |
| Soups, sauces, seasonings and flavorings |  |  |
| Beverages |  |  |
| Infant and paediatric feeds and foods |  | Some infant feeds |
| Therapeutic/special/diet products |  |  |
| Miscellaneous | Eggnog, liqueur with cream | Tea |
